# Adherence to Exercise in People with Lung or Head and Neck Cancer: Self-Reported Symptoms and Motivation During Cancer Treatment Need to Be Considered

**DOI:** 10.3390/jcm13206267

**Published:** 2024-10-21

**Authors:** Matheus Pedroso, Isis Grigoletto, Letícia Oliveira, Sarah Martins, Lara Costa, Karina Pozo, Paloma Borges, Livia Regio, Isabela Duarte, Vinicius Cavalheri, Ercy Ramos

**Affiliations:** 1 Department of Physiotherapy, Faculty of Science and Technology, São Paulo State University (UNESP), Presidente Prudente 19060-900, São Paulo, Brazil; matheus.a.pedroso@unesp.br (M.P.); isis.grigoletto@unesp.br (I.G.); leticia.vilas@unesp.br (L.O.); sh.martins@unesp.br (S.M.); lara.paschoal@unesp.br (L.C.); karina.pozo@unesp.br (K.P.); paloma.borges@unesp.br (P.B.); livia.regio@unesp.br (L.R.); isabela.duarte@unesp.br (I.D.); ercy.mara@unesp.br (E.R.); 2 Curtin School of Allied Health, Faculty of Health Sciences, Curtin University, Perth, WA 6102, Australia; 3 Allied Health, South Metropolitan Health Service, Murdoch, WA 6150, Australia; 4 Regional Cancer Hospital, Presidente Prudente 19013-050, São Paulo, Brazil; 5 Onco Care, OncoClínicas, Presidente Prudente 19053-240, São Paulo, Brazil

**Keywords:** neoplasms, head and neck neoplasms, lung neoplasms, exercise, motivation, symptom assessment, symptom burden

## Abstract

**Objectives**: Symptoms and motivation may impact adherence to home-based exercise training programs (HETP) during cancer treatment (CT) for lung or head and neck cancer. This study aimed to identify self-reported symptoms and their frequency, as well as motivation towards an HETP during CT for primary lung or head and neck cancer. Associations between symptoms and motivation with HETP adherence were also investigated. **Methods**: Participants underwent CT combined with an HETP that included aerobic (walk-based) and resistance training (Theraband^®^). Weekly assessment was conducted using a questionnaire developed by the researchers, evaluating the presence of symptoms. A scale (0 to 10) was used to assess motivation towards the HETP. Adherence was defined as the ratio between HETP sessions completed vs. the number prescribed. Symptom frequency was recorded as the number of weeks a symptom was experienced. Linear regression was used to explore associations. **Results**: Twenty-four participants were included (61 ± 7 yr; 21 males; head and neck cancer *n* = 18; median treatment duration: 9 [7 to 11] weeks). The most commonly reported symptoms were fatigue (33%), malaise (24%) and dysphagia (23%). Average score for motivation to exercise was 6.4 ± 2.0. Adherence to the HETP was 47%. Malaise was associated with reduced adherence to HETP (*p* = 0.002), explaining 35% of the variance. Motivation was associated with increased adherence (*p* = 0.008), explaining 28% of the variance. **Conclusions**: Fatigue, malaise and dysphagia were among the most frequently reported symptoms during treatment. Malaise and self-motivation to exercise can significantly influence adherence to HETPs. Symptom and motivational support might be necessary when implementing HETPs during CT.

## 1. Introduction

Cancer remains a leading cause of death globally, with lung cancer being the second most diagnosed and leading cause of death in cancer [1]. Although head and neck cancers have lower mortality rates, their growing incidence and shared risk factors with lung cancer warrant equal attention [2]. These shared risk factors include tobacco smoking, occupational exposures, air pollution and alcohol consumption [2,3].

These risk factors may also complicate treatment due to their association with other health problems, which may be aggravating in patients who already tend to show lower functional capacity and quality of life during treatment [4,5,6,7,8].

Treatment for these cancers can include surgery, chemotherapy and radiotherapy. The type and duration of treatment are tailored to the disease and patients’ characteristics, including whether the cancer is at an early or advanced stage and whether the patient is fit or not for surgery [9,10]. These treatments, while effective, can also cause adverse symptoms, such as fatigue [11,12], sleep disturbances [13], emotional stress, anxiety, depression [14,15], loss of muscle mass and pain [16,17].

Previous systematic reviews suggest that exercise training programs can be implemented as a complementary treatment for patients undergoing cancer treatment in a safe and effective manner, promoting improvements in treatment completion rates [18], functional capacity [18,19,20,21], psychosocial function [22] and quality of life [20,22,23].

Home-based aerobic and resistance exercise, specifically, is relatively novel and not yet deeply explored in cancer treatment. These interventions present a promising option for cancer care, with a systematic review of nine studies showing that, despite the methodological limitations of included studies, home-based exercise is feasible in cancer survivors, and may provide benefits in peripheral muscle strength, exercise capacity and quality of life [24].

When implemented during the period of chemotherapy, radiotherapy or chemoradiotherapy, home-based exercise shows great potential, as it can overcome common limitations and barriers to usual supervised group-based exercise interventions in this population (e.g., risk of infections, limited capacity to travel to an exercise facility, limited time, elevated cost and lack of specialized centers for exercise in cancer patients) [24,25].

Although exercise training is effective at improving health outcomes in people undergoing cancer treatment, symptoms experienced during that period can be considerably complex and severe, potentially impacting adherence to exercise [26,27,28,29,30]. Of note, detailed information on different symptoms experienced by cancer patients (and frequency of these symptoms) in studies of home-based exercise training has not been reported [31,32,33,34,35]. Therefore, the impact of symptoms on adherence to home-based exercise training is unknown.

Intrinsic motivation to exercise is another important factor not explored in the exercise oncology literature that may impact on adherence to home-based exercise, especially during cancer treatment [24]. Notably, motivation to exercise has been shown to be associated with exercise adherence in other populations [36]. Gaining insight into whether symptoms and motivation to exercise affect adherence to home-based exercise programs would enable health professionals to more effectively customize these interventions for people with cancer.

The aims of this study were, in individuals diagnosed with lung or head and neck cancer undergoing a home-based exercise training program during chemotherapy and/or radiotherapy, to explore the frequency of weekly self-reported symptoms and motivation to exercise and to explore associations between these variables and adherence to the home-based exercise training program.

## 2. Materials and Methods

This study is a secondary analysis of a randomized controlled trial (RCT) [37] conducted in a private clinic, OncoClínicas, and a public hospital, Cancer Hospital of Presidente Prudente, both located in Presidente Prudente, São Paulo, Brazil. The trial has received approval from the health research ethics committee of the Regional Cancer Hospital of Presidente Prudente (HRCPP; 26123119.5.0000.8247), which complies with the Declaration of Helsinki. The trial was also registered in the Brazilian Clinical Trials Registry (ReBEC) (RBR-5cyvzh9). Written, informed consent to participate was obtained from all participants.

In summary, the RCT included participants diagnosed with lung or head and neck cancer undergoing chemotherapy and/or radiotherapy who were randomized to either an intervention group (IG) or a control group (CG). Those allocated to the IG underwent a home-based exercise training program during cancer treatment, whereas those allocated to the CG were asked to maintain their usual daily life activities during cancer treatment. The full details of the methods of the RCT have been described elsewhere [37]. In this secondary analysis, only data from the participants allocated to the IG were included. Participants were contacted via a phone call weekly during the study, and specific questions on symptoms they experienced and their motivation to complete the prescribed home-based exercise sessions were addressed.

Eligible participants were identified and recruited from the outpatient clinic and hospital before cancer treatment commencement. Recruitment for this study took place between 09/2020 and 12/2023. The inclusion and exclusion criteria were described in the published protocol [37]. Briefly, the inclusion criteria were as follows: (1) adults >18 years of age; (2) diagnosis of primary lung or head and neck cancer; (3) capable of performing the tests in the initial assessment; (4) capable of understanding and performing the exercise training program adequately; and (5) undergoing the prescribed chemotherapy and/or radiotherapy treatment. The exclusion criteria were as follows: (1) brain or bone metastases; (2) withdrawal from cancer treatment at any time during the study; (3) those on palliative care.

### 2.1. Home-Based Exercise Training Program Protocol

The prescribed home-based exercise training program included both aerobic and resistance exercises. Participants received weekly training programs with comprehensive instructions and visual illustrations on the exercises prescribed. The protocol [37] was developed by the research group and pilot-tested with a small cohort to ensure comprehensiveness.

The initial exercise prescription included walking daily for at least 20 min [23] and performing elbow flexion, knee flexion and knee extension exercises twice a week [19] using moderate-resistance TheraBands^®^ (Hygenic Corporation, Akron, OH, USA—Sourced in Brazil) (green color). The exercises performed were recorded on a daily log that was returned to the researchers on a weekly basis.

The exercise training program commenced the week before the start of cancer treatment and continued throughout it, ending two weeks after completion of cancer treatment. The modified Borg dyspnea scale [19,20] was used to progress the aerobic and resistance exercises. Participants were instructed to exercise at an intensity between 4 and 6 on the Borg dyspnea scale, and when the walking intensity was reported as <4 on the Borg scale, five extra minutes of walking were prescribed. For the resistance exercises, when the intensity was reported as <4 on the Borg scale, either one extra set or five extra repetitions were added [37].

### 2.2. Initial Assessments

Functional exercise capacity was assessed through the six-minute walk test, in accordance with the guidelines established by the American Thoracic Society and European Respiratory Society [38] and the 1 min sit-to-stand test. Reference values for the Brazilian population were used [39]. Symptoms of anxiety and depression previous to starting cancer treatment were assessed via the Hospital Anxiety and Depression Scale [40].

### 2.3. Assessing Adherence to Home-Based Exercise Training

Adherence was measured via the ratio between completed and prescribed exercise sessions and expressed as a percentage. These data were extracted from the daily logs that were filled out by the participants.

### 2.4. Assessing Self-Reported Symptoms’ Frequency

Self-reported symptoms were assessed weekly throughout the intervention window via phone calls. This approach was chosen due to its practicality, accessibility and consistency in weekly data collection when considering individuals undergoing chemotherapy and/or radiotherapy, who might face limitations in capacity to travel, time constraints or symptomatic burden that would make in-person interviews difficult [24,25].

To assess these self-reported symptoms, a questionnaire was developed by the researchers and pilot-tested with a small sample of participants to ensure clarity and accuracy in their reported symptoms. During the initial assessment, participants were introduced to the structure and application of the questionnaire. The questionnaire included a range of possible symptoms frequently reported in literature [8,23,26,27], including fatigue, malaise, dysphagia, joint pain, muscle pain, headaches, chest pain, throat pain, anxiety, sadness and irritability. Participants were asked to indicate whether or not they had experienced each of the symptoms in the past seven days with a “yes” or “no” answer.

### 2.5. Assessing Self-Reported Motivation

During the weekly phone call, after assessing symptoms, motivation to perform the prescribed exercise sessions was also assessed. This was facilitated through a scale ranging between 0 (zero—no motivation at all) and 10 (ten—very, very motivated).

### 2.6. Statistical Analysis

Data analysis was performed using the Statistical Package for Social Sciences (SPSS) version 29.0 (IBM Corp, Armonk, NY, USA) and R v4.3.1 (R Foundation, Vienna, Austria). Figures were made using R v4.3.1, RStudio v2023.9.1.494 (Posit PBC, Boston, MA, USA) and GraphPad Prism 8.0.2 (GraphPad Software, Boston, MA, USA). Data distributions were assessed by the Shapiro–Wilk test. Data were reported for the total sample, as well as for the subgroups of people with lung cancer or head and neck cancer; these subgroups were compared using the Mann–Whitney or Student’s *t* tests, according to data distribution.

Adherence to the home-based exercise training program was expressed as a percentage, based on the mean ratio between completed and prescribed sessions for all participants. The proportion of symptoms reported are expressed as the percentage of weeks in which a symptom was present in relation to the number of weeks each participant was under CT (e.g., if a participant reported experiencing fatigue in 4 weeks and was in CT for 19 weeks, the frequency of fatigue was calculated as 4/19 = 0.21, or 21%). Motivation is expressed as a mean ± SD based on all participants’ assessments throughout CT.

Data (mean ± standard error of the mean) regarding the weekly adherence to the home-based exercise training program, weekly scores for motivation towards the program and the total number of weekly symptoms reported during the first weeks of treatment were plotted using GraphPad Prism 8.

Linear correlations between adherence to the home-based exercise training program, the proportion of weeks a symptom was reported and the weekly scores for motivation towards the program across the whole intervention window were tested using Pearson’s or Spearman’s coefficients. Linear regression models were used to determine the association between adherence to the home-based exercise program, the proportion of weeks a symptom was reported, specifically malaise, which showed statistically significant linear correlation results, and motivation towards the exercise program.

The sample size for this study was based on the availability of eligible participants during the recruitment phase (n = 24). Post-hoc power analysis was conducted for each linear regression model to ensure sufficient power in each model, considering a power threshold of 80%; all models presented >80% power to detect effects of symptoms and motivation on adherence. Effect size was assessed for each linear regression model via Cohen’s f^2^. Statistical significance was set at 5%.

## 3. Results

**Figure 1:**
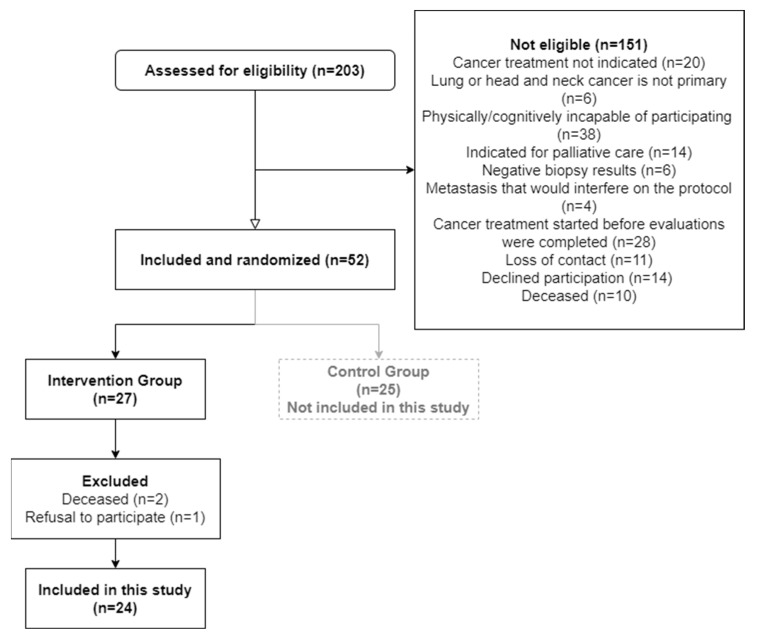
Study flowchart.

A total of 24 participants were included in the analysis: 6 with lung cancer and 18 with head and neck cancer. The study’s flowchart is present in Figure 1. Participants’ characteristics are presented in Table 1. The median intervention duration was 9 (7 to 11) weeks.

### 3.1. Adherence to Home-Based Exercise Training

For the total sample, adherence to the home-based exercise training program was 47%. Participants with lung cancer completed 37% of the prescribed sessions, whereas those with head and neck cancer completed 50%. There were no statistically significant differences in adherence to the home-based exercise training program between cancer types.

### 3.2. Symptoms and Motivation

**Figure 2:**
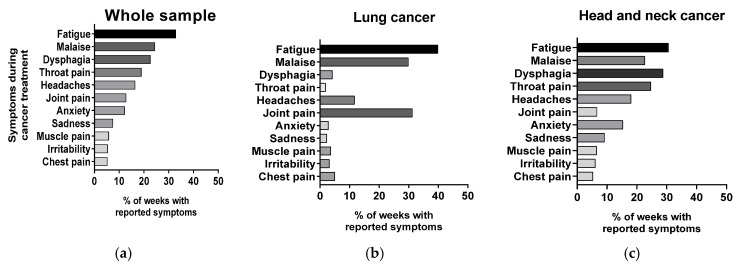
Frequency of the symptoms reported by participants: (**a**) all participants; (**b**) participants with lung cancer; (**c**) participants with head and neck cancer.

Symptoms reported as well as their proportion in relation to the total number of CT weeks are presented in Figure 2. The most frequently reported symptom was fatigue (33% of weeks during CT), followed by malaise and dysphagia (24% and 23% of weeks during CT, respectively). The only statistically significant differences in symptoms between cancer types were throat pain, more frequent in head and neck cancer (25% vs. 2%; *p* = 0.033), and joint pain, more frequently reported in lung cancer (31% vs. 7%; *p* = 0.033).

For the total sample, the mean score for motivation towards the home-based exercise training program was 6.4 ± 2.1. The mean motivation scores of participants with lung cancer and those with head and neck cancer were 6.6 ± 2.2 and 6.4 ± 2.2, respectively. There were no statistically significant differences in motivation between cancer types.

**Figure 3:**
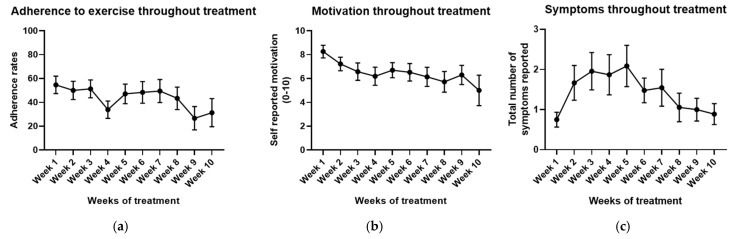
Adherence, motivation and general symptom variations for the first ten weeks of treatment for all participants: (**a**) variation in adherence to exercise; (**b**) variation in motivation to exercise; (**c**) variation in total number of reported symptoms per week. Data expressed as mean ± SEM.

Weekly adherence to the home-based exercise training program, weekly scores for motivation towards the program and the total number of weekly symptoms reported during the first ten weeks of treatment are presented in Figure 3.

### 3.3. Associations Between Adherence to Home-Based Exercise, Motivation to Exercise and Self-Reported Symptoms During Treatment

Linear regression models were used to verify associations between the adherence to the home-based exercise training program, the proportion of symptoms reported throughout CT and motivation towards the program. Only symptoms with statistically significant results will be included on the following tables. Table 2 presents simple linear regression models (i.e., model 1—malaise; model 2—motivation), and Table 3 presents a multiple linear regression model containing both of these variables.

When considered individually, malaise was significantly associated with exercise adherence, explaining 35.2% of its variance. Each unit of malaise decreased adherence by approximately 0.69 units, with an effect size (Cohen’s f^2^) of 0.5, which is considered large. Motivation was also significantly associated with adherence, explaining 27.8% of the variance. Each unit of motivation increased adherence by approximately 7.2 units, with an effect size of 0.4, also considered large. When both malaise and motivation were included in a multivariate model, they explained an even higher proportion (47.4%) of the variance in exercise adherence during treatment with an effect size of 0.9, considered large.

## 4. Discussion

Our study demonstrated the following results in people with lung or head and neck cancer undergoing a home-based exercise training program during chemotherapy and/or radiotherapy: (i) Fatigue was the most frequently reported symptom, present in 33% of treatment weeks. Fatigue was followed by malaise and dysphagia, present in 24% and 23% of treatment weeks, respectively. (ii) Adherence to the home-based exercise training program was 47%. Finally, (iii) malaise and motivation to exercise were significantly associated with adherence to the home-based exercise training program.

Regarding symptoms, our findings align with previous literature that reported fatigue as being a predominant symptom during cancer treatment [41]. However, previous studies simply reported the prevalence of symptoms amongst cancer patients at a specific timepoint [41], rather than a weekly evaluation of symptoms throughout cancer treatment, as conducted in our study.

Our 47% adherence to the home-based exercise training program is somewhat lower than the adherence reported in most previous studies (ranging between 65% and 88%) [31,34,42,43]. This discrepancy can be related to multiple factors, including the fact that in most of these studies, participants had less severe cancers. Other factors that could have led to differences in adherence include the duration and intensity of interventions, tumor location, participants’ interest in exercise, their preference regarding the specific protocols implemented in this and other studies or simply how adherence is assessed. To our knowledge, our study was the first to focus on home-based exercise training for lung or head and neck cancer, which are devastating cancers when it comes to burden on patients. Also, our study assessed adherence through training diaries, similarly to methods employed in previous studies [18,33,34,43]. However, unlike in some of these studies, we measured adherence as the percentage of completed sessions by all participants, rather than the percentage of participants who fully adhered to the prescribed program. The assessment and reporting of training adherence in home-based interventions is important and recommended [24]. However, guidelines on how this assessment and reporting should be performed are necessary, as standardization in this field would be beneficial.

Regarding what might influence adherence to exercise, malaise and motivation to exercise were significantly associated with adherence to home-based exercise, with each unit of malaise decreasing roughly 0.7 units of adherence, and each unit of motivation increasing roughly 7 units of adherence. This suggests that symptoms can significantly hinder adherence and, at the same time, patients’ intrinsic motivation may be a decisive factor in maintaining a home-based exercise regimen. A recent review showed that low self-motivation is one of the main barriers to adherence to home-based exercise interventions [44]; even so, motivation to exercise is still not commonly monitored throughout cancer treatment or in research related to home-based exercise training for cancer patients [24,36]. Symptoms were also relevant and should be more frequently measured [26,27]. Fatigue, depression and other symptoms are common factors known to reduce adherence to usual exercise interventions, and they may even play a more significant role in adherence to unsupervised home-based exercise training programs [44,45,46,47,48].

Home-based exercise training offers the possibility of overcoming barriers to attendance and adherence to center-based exercise training in people undergoing CT. Some of these barriers include risk of infection, limited capacity to travel frequently, limited time and lack of specialized exercise centers [24,25]. Our study contributes to the literature by demonstrating that symptoms experienced during cancer treatment and motivation to exercise play a significant role in adherence to home-based exercise training. Regular assessment and appropriate management of both symptoms and motivation to exercise could aid healthcare professionals in designing, prescribing and maintaining exercise programs (whether home-based or in exercise centers) for cancer patients, particularly for lung or head and neck cancers. By taking these factors into consideration, a multidisciplinary team could enhance adherence rates and possibly improve overall outcomes. For example, physicians can manage symptoms like malaise, physiotherapists can adjust exercise intensity or type based on symptom burden and behavioral therapists or psychologists can offer motivational support and help address barriers to exercise [26,27,28,36,46,49].

Future studies are necessary to better understand which patients are more likely to experience specific symptoms or lower motivation to exercise. Individual characteristics and preferences [50] should be considered, and patient profiling would be ideal to investigate [51]. Such studies should also explore how multidisciplinary interventions can minimize some of these barriers [52] and investigate whether pre-treatment educational interventions aimed at addressing (i) symptoms and behavior toward symptoms and (ii) the importance and benefits of exercise training in people with cancer could help increase adherence to exercise [29,30,36] and improve outcomes.

To our knowledge, this is the first study to measure weekly self-reported symptoms and motivation to exercise in individuals undergoing CT and explore how these factors are associated with adherence to a home-based exercise training program designed to improve exercise capacity and physical activity levels in this population. While providing valuable information, this study’s limitations should be taken into consideration. The home-based exercise training program implemented consisted of walking and TheraBand exercises. These were chosen based on their practicality and accessibility; however, this protocol may not be suitable for all participants, especially when considering their different physical abilities and personal preferences. Tailoring the exercise program based on patients’ physical capacity and preference could help improve adherence. Future studies would benefit in exploring more personalized exercise interventions.

Also, the interviews were conducted using a non-validated questionnaire developed by the researchers to assess symptoms and motivation, while the questionnaire was based on previous studies and pilot-tested to ensure its clarity and refine items, it was not formally tested for reliability and validity. Future studies should employ validated, or validate new, practical symptom-tracking instruments, which could provide more robust evidence to support our findings. The data were collected through phone calls. This approach is practical, accessible and in some cases necessary for individuals undergoing CT. Although participants were introduced to the structure and application of the questionnaire beforehand, this method, coupled with possible recall bias, may affect the reliability of the data. Future research would greatly benefit from more frequent data collection methods, possibly through daily symptom diaries, daily interviews or digital symptom tracking via a mobile application. These approaches, although possibly difficult to implement in this population, could minimize recall bias and improve data accuracy.

Another potential limitation includes the different cancer types. While no statistically significant differences were found in exercise adherence and motivation, two symptoms (joint and throat pain) showed significant differences between the subgroups. This difference in symptom profile and treatment regimens could introduce bias, possibly influencing adherence to exercise in other cases, with different exercise protocols or cancer types. Consequently, the generalizability of these findings may be limited. Future studies with larger and more homogenous populations are needed to corroborate these results and explore how they would apply to a broader cancer population.

Despite its limitations, this study is the first to systematically assess weekly symptom fluctuations and their impact on exercise adherence during cancer treatment, providing valuable insights for future research and clinical practice.

## 5. Conclusions

People with lung or head and neck cancer undergoing a home-based exercise training program during chemotherapy and/or radiotherapy reported fatigue as the most frequent weekly symptom, followed by malaise and dysphagia. Their adherence to the home-based exercise training program was 47%, and malaise and motivation to exercise were significantly associated with adherence to the home-based exercise training program. Our findings highlight the need for more proactive and frequent measurement of symptoms and motivation to exercise in cancer care, as well as better management of symptoms and motivation. Future research should investigate multidisciplinary approaches, including behavioral and motivational strategies, to assist in symptom management and improve adherence to home-based exercise training and achieve better outcomes for people undergoing CT.

## Figures and Tables

**Table 1:** Characteristics of participants (*n* = 24).

	Total Sample (*n* = 24)	Lung Cancer (*n* = 6)	Head and Neck Cancer (*n* = 18)
Anthropometric characteristics			
Gender, male (%)	21 (88%)	3 (50%)	18 (100%)
Age (years)	61 ± 7	58 ± 4	62 ± 8
BMI (kg/m^2^)	24 ± 4	27 ± 4	23 ± 4
Cancer treatment			
Chemotherapy	7 (29%)	6 (100%)	1 (6%)
Radiotherapy	7 (29%)	0 (0%)	7 (39%)
Chemotherapy + Radiotherapy	10 (42%)	0 (0%)	10 (56%)
Six-minute walk test			
Six-minute walk distance (m)	519 ± 79	495 ± 59	527 ± 84
Six-minute walk distance (% predicted)	90 ± 14	91 ± 11	90 ± 16
1 min sit to stand test (repetitions)	24 ± 7	22 ± 4	24 ± 7
Hospital Anxiety and Depression—Anxiety (score)	6 ± 3	6 ± 5	5 ± 3
Hospital Anxiety and Depression—Depression (score)	5 ± 4	6 ± 6	5 ± 4

kg: kilograms; m: meters; %: percentage. No statistically significant differences were found between cancer types for the variables above (*p* > 0.05).

**Table 2:** Simple linear regression results. Associations between exercise adherence, symptoms and motivation.

Variables	Beta (95% CI)	*p*-Value
Malaise	−0.594 (−1.11, −0.28)	0.002 *
Motivation	0.528 (2.08, 12.35)	0.008 *

Beta: Standardized coefficients; 95% CI: 95% confidence interval; *p*-value: * *p* < 0.05.

**Table 3:** Multiple linear regression results. Associations between exercise adherence, symptoms and motivation.

Variables	Beta (95% CI)	*p*-Value
Constant	-	0.134
Motivation	0.370 (0.29, 9.8)	0.039 *
Malaise	−0.470 (−0.96, −0.14)	0.011 *

Beta: Standardized coefficients; 95% CI: 95% confidence interval; *p*-value: * *p* < 0.05.

## Data Availability

The datasets presented in this article are not readily available because the data are part of an ongoing study. Requests to access the datasets should be directed to the corresponding author.
